# LONGITUDINAL ASSOCIATIONS OF HEARING IMPAIRMENT WITH SOCIAL ISOLATION AMONG BLACK AND WHITE OLDER ADULTS

**DOI:** 10.1093/geroni/igae098.4017

**Published:** 2024-12-31

**Authors:** Mary Thoma, Erin Ferguson, Jacqueline Torres, Nicole Armstrong, Kristine Yaffe, Willa Brenowitz

**Affiliations:** University of California San Francisco, San Francisco, California, United States; University of California, San Francisco, San Francisco, California, United States; University of California San Francisco, San Francisco, California, United States; Warren Alpert Medical School of Brown University, Providence, Rhode Island, United States; University of California San Francisco, San Francisco, California, United States; Kaiser Permanente, Portland, Oregon, United States

## Abstract

Social isolation has been declared an epidemic by the U.S. Surgeon General. Hearing impairment (HI) may be an important risk factor for isolation among older adults. Studies of HI and isolation have been inconclusive and use primarily cross-sectional and self-reported data. We used data from the HealthABC study (N=2227 Black and White adults aged 70-79 at recruitment) to examine associations of baseline objective HI (average pure-tone audiometry >40 dB, with and without use of hearing aids) with frequency of contact with family and friends (< weekly vs. at least weekly), depressive symptoms (CESD-10), and loneliness (frequency in past week; 1-4) across 6 visits (6-7 years). We used mixed effects models adjusted for demographic and clinical variables. Untreated objective HI was associated with increased odds of < weekly contact with friends (OR=1.47; 95% CI: 1.08,2.00) and more depressive symptoms (B=0.64; 95% CI: 0.26,1.01) across follow-up, but was not with frequency of family contact or loneliness. Objective HI treated with hearing aids was not associated with any outcome. We additionally found that self-reported HI (“hearing limits personal/social life”) was associated with more depressive symptoms (B=1.49; 95% CI: 1.03,1.95) and loneliness (B=0.09; 95% CI: 0.01,0.16) across follow-up. Our results provide novel evidence that untreated objective hearing loss is associated with social isolation and subjective HI is associated with psychosocial wellbeing. Treatment for HI may reduce the likelihood of social isolation.

